# (In)Consistent Performance Feedback and the Locus of Search

**DOI:** 10.1177/01492063231185519

**Published:** 2023-07-27

**Authors:** Thomas Keil, Evangelos Syrigos, Konstantinos C. Kostopoulos, Felix D. Meissner, Pino G. Audia

**Affiliations:** University of Zurich; 30944 Zurich University of Applied Sciences; 69000 University of Piraeus; University of Zurich; 3728 Dartmouth College

**Keywords:** behavioral theory of the firm, multiple goals, locus of search, inconsistent performance feedback, self-enhancement

## Abstract

Despite the prevalence and importance of multiple goals for organizations, research on how organizations respond to performance on multiple goals continues to be limited and has examined only search intensity as the focal response, ignoring that search may occur in different locations. We extend the research on multiple goals by developing and testing novel theory on the relationship between performance feedback on multiple goals and the locus of search. Drawing upon the behavioral theory of the firm and using panel data from global pharmaceutical firms, we first show that when performance is below aspirations on a primary goal, a firm's propensity to engage in distal search increases along both the technological (i.e., familiar vs. unfamiliar search) and the organizational dimension (i.e., internal vs. external search). However, building on more recent literature that points to the need to consider multiple goals of unequal importance and, specifically, the self-enhancement perspective, we argue and find that performance on a secondary goal modifies this pattern, particularly when performance on a primary goal is unsatisfactory. Under feedback inconsistency, where performance on a primary goal is low but performance on a secondary goal is high, decision-makers decrease distal search to both unfamiliar technological areas and areas external to the organization. Our theory and findings highlight the importance of performance feedback regarding multiple goals in regulating the key locus of search choices and extend research on self-enhancement and learning from performance feedback.

## Introduction

Starting from Cyert and March's (1963) seminal book, organizational scholars have accumulated a considerable body of work that documents the influence of performance in relation to an aspiration level on a wide range of organizational responses. Although multiple goals are a permanent fixture of organizations (Ethiraj & Levinthal, 2009; Levinthal & Rerup, 2021), the preponderance of work on the influence of performance on organizational responses bypassed this critical aspect of organizations by assuming that decision-makers pick a particular (typically financial) goal to focus their attention on and then stick to such an orientation (Cyert & March, 1963; Greve, 2008). Studies of the impact of return on assets (ROA) are the most prominent example of this research approach (see, Posen, Keil, Kim, & Meissner, 2018 for a review). It is only recently that we are witnessing a gradual shift toward the examination of the influence of multiple goals, and particularly of situations where performance on such goals diverges, thus leaving open different possible interpretations of overall performance (e.g., Audia & Greve, 2021; Gaba & Greve, 2019; Hu & Bettis, 2018; Mazzelli, Nason, De Massis, & Kotlar, 2019). More importantly, the limited research on multiple goals has examined search intensity as the focal response, ignoring that search may occur in different locations.

The goal of this study is, therefore, to contribute to this body of work by examining the influence of performance on multiple goals on search, a central concept in research on the effect of performance on organizational behavior (Greve, 2003b), and, in particular, the locus of search (i.e., local vs. distal search). Single-goal studies of performance and search have focused on Research and Development (R&D) intensity as a proxy for search, with the key prediction and finding being that low performance on a primary goal such as ROA increases R&D intensity and thus evidences an increase in overall search (Chen, 2008; Greve, 2003a; Lucas, Knoben, & Meeus, 2018; O’Brien & David, 2014). Other studies examining multiple goals (Audia & Brion, 2007; Kostopoulos, Syrigos, & Kuusela, 2023) suggest that, when performance is low on a primary goal but high on a secondary goal, organizations may not increase the intensity of search activities as predicted by Cyert and March (1963). Yet, pertinent literature is silent about the influence of this type of inconsistent performance regarding multiple goals on how organizations regulate the locus of search decisions and, in particular, their propensity to engage in (more or less) distal search. Distal search is of critical importance to organizations and has been extensively examined in the technological literature (Laursen, 2012). An organization may search more or may search less, but the search that it initiates may take place in the proximity of familiar technological areas or inside the organization (i.e., local search) or, alternatively, may occur in unknown technological areas or outside the organization (i.e., distal search). In this study, therefore, we extend previous work on multiple goals by addressing the following questions: Does low performance on a primary goal lead to an increase in distal search? Does high performance on a secondary goal, when performance on a primary goal is also low, reduce distal search?

To address these research questions, we integrate insights from the original formulation of the behavioral theory of the firm (BTOF) laid out by Cyert and March (1963) and later elaborations that give greater emphasis to the influence of multiple goals (e.g., Audia & Greve, 2021; Gaba & Greve, 2019; Hu & Bettis, 2018). A long-held assumption rooted in Cyert and March (1963) is that boundedly rational decision-makers oriented to problem-solving handle the cognitive demands that stem from multiple goals by sequentially allocating attention, focusing first exclusively on primary goals that are highest in a hierarchy of importance and then moving down the goal hierarchy (i.e., to secondary goals), as the goal that has higher importance is met. Drawing upon this perspective, in our first hypothesis, we argue that organizations will respond with more distal search (Baum, Rowley, Shipilov, & Chuang, 2005) when performance on a primary goal is below the aspiration level, while performance on a secondary goal does not influence where (locally or distally) search is conducted unless performance on the primary goal is satisfactory.

However, later theorizing that focuses on multiple goals points to a contrasting scenario. When decision-makers experience multiple goals of unequal importance, they may self-enhance (Audia & Brion, 2007) and focus their attention on the goals that show positive outcomes, irrespective of whether they are the most important. Prior related research (Kostopoulos et al., 2023) has argued that when performance on a primary goal is low, performance on a secondary goal that exceeds an aspiration level leads to a reduction in the intensity of search activities. We add a new insight, namely that the propensity to self-enhance, enabled by the ambiguity that arises when performance on a secondary goal is high and performance on a primary goal is low, will manifest itself also in important locus of search choices. Specifically, we argue that decision-makers who are motivated to self-enhance prefer to direct search in more familiar technological areas and to keep search internal to the organization, as such search reinforces trust in their previous choices and skills and, thus, supports their positive self-image. They shy away from searching in unfamiliar areas or outside the organization because these two forms of distal search can be perceived as casting doubt on their ability to generate plausible solutions with their current set of knowledge and skills.

We examine the influence of performance on multiple goals on distal search using a unique sample of global incumbent pharmaceutical firms with a portfolio of clinical trials regulated by the U.S. Food and Drug Administration (FDA) during the period 1993 to 2021. Specifically, we examine how decision-makers respond to portfolio-level performance feedback regarding multiple stages of the clinical trials process. We found that, taken by itself, performance relative to aspirations on a primary goal influences a firm's propensity to search distally along two independent dimensions: the technological dimension, which refers to familiarity with the firm's technological areas, and the organizational dimension, which refers to knowledge and expertise located within or outside the firm (Rosenkopf & Nerkar, 2001; Rothaermel & Alexandre, 2009). Specifically, when the rate of successful late-stage clinical trials (the primary goal) is below aspirations, firms increase their propensity to select drug candidates targeted at disease areas that are unfamiliar to the firm as well as drug candidates from outside the firm. However, consistent with our prediction, our findings also indicate that when above-aspiration performance on a secondary goal (i.e., the rate of successful early-stage clinical trials) is considered, this pattern reverses. Under feedback inconsistency, where performance on a primary goal is low but performance on a secondary goal is high, decision-makers decrease distal search along both the technological and the organizational dimensions. Distal search increases only when performance is consistent and below aspirations on both goals.

Our arguments and results make two significant contributions to research on performance feedback. Specifically, we develop and test novel theory on how performance feedback on multiple goals that follow a hierarchy of importance influences how organizations regulate the locus of search, particularly distal search. We show that poor performance on a primary goal may signal a serious problem that “rules out the space of solutions local to currently employed activities” (Posen et al., 2018: 237) and directly triggers distal solution search, but only if performance feedback on a secondary goal also exhibits performance below aspirations. By contrast, performance below aspirations on a primary goal and performance above aspirations on a secondary goal prompt decision-makers to redirect search to less distal technological and organizational dimensions. Our arguments and findings are important because prior research on multiple goals has exclusively considered search intensity, ignoring that search may occur in different locations.

Our research also makes a novel contribution to studies of self-enhancement and learning from performance feedback. Prior work has argued that self-enhancement motivates decision-makers to reduce search in response to performance feedback (Blagoeva, Mom, Jansen, & George, 2020; Jordan & Audia, 2012). To our knowledge, this is the first study to propose that the self-enhancement motive may also account for variations in the locus of search. Indeed, our theory and findings suggest that, in situations of feedback inconsistency regarding multiple goals, self-enhancing decision-makers may experience greater latitude to choose whether to respond by directing search inside the organization or to more familiar technologies instead of only reducing the intensity of search altogether as previous research would predict. Self-enhancement, therefore, may not only lead to a (re)prioritization of goals, by focusing attention on a secondary goal that shows high performance, but also to a (re)prioritization of the locus of search choices that decision-makers are willing to pursue to protect their self-image. In this way, this study opens a new line of research that examines the influence of self-enhancement on learning from performance feedback by suggesting that the desire to maintain and boost a positive self-image may also be fulfilled by selecting specific loci of responsive actions.

## Theory and Hypotheses

Most research on the relationship between performance and organizational responses has assumed that organizations focus only on a single goal at any time. The goal that has dominated researchers’ attention is ROA (Greve, 2003b). But while ROA is often the highest-level goal to which senior executives allocate their attention, more recent work has started to recognize a wide range of other goals that may coexist with profitability, such as productivity (Mazzelli et al., 2019), size (Greve, 2008), status (Baum et al., 2005), or safety (Gaba & Greve, 2019). Such multiple goals are typically arranged in a hierarchy of importance where some goals are more important than others. The prevailing assumption is that in the context of for-profit organizations, profitability metrics and their close correlates, such as market share and sales growth, are at the top of the hierarchy of goal importance because of their commonly agreed-upon nature (Shinkle, 2012). As Rowley, Shipilov and Greve (2017: 819) note, “If the firm does not generate satisfactory financial results, it will simply be not viable for very long.” Indeed, some studies that have examined multiple goals support this view, suggesting, for example, that goals that have a more distant impact on profitability—such as growth (Greve, 2008) and corporate governance ratings (Rowley, Shipilov, & Greve, 2017)—occupy a lower position in the hierarchy of goal importance.

Once the existence of multiple goals of varying importance is acknowledged, the question arises of how performance on these goals affects organizational responses. The traditional view in the literature follows Cyert and March's (1963) sequential attention approach to suggest that organizations attend first to performance signals on primary goals, as these are perceived as more salient, and, once these goals are fulfilled, performance feedback on secondary goals is activated (Greve, 2008). Yet, recent studies have started to highlight that, in many situations, decision-makers may need to process multiple goals simultaneously rather than sequentially (Audia & Greve, 2021; Gaba & Greve, 2019; Hu & Bettis, 2018). In these instances, they are confronted with both consistent and inconsistent performance feedback (Audia & Brion, 2007), on which they rely to determine whether current courses of action regarding primary and secondary goals denote success or failure.

Incorporating multiple goals raises the question of how organizations respond to inconsistent performance signals. Research that emphasizes a self-enhancement approach suggests that decision-makers may consider multiple goals simultaneously and may first and foremost be motivated to maintain a positive image and focus on information that emphasizes high performance (Audia & Brion, 2007; Jordan & Audia, 2012). Because they care about maintaining a positive image (Audia & Brion, 2007), decision-makers primarily responsible for the outcomes measured using multiple goals will capitalize on the ambiguity generated by inconsistent performance feedback to assign greater weight to the goals that display positive outcomes. Such a favorable assessment of performance, whereby low performance on a primary goal is de-emphasized so that greater consideration is given to high performance on a secondary goal, has been found to reduce the propensity to make changes (Audia & Brion, 2007; Kostopoulos et al., 2023), and is hypothesized to reduce the intensity of search (Jordan & Audia, 2012).

While prior research on multiple goals has added much to our understanding of how consistent and inconsistent feedback regulates the intensity of search, it has remained silent regarding a second, equally important dimension of search, namely the locus of search (Greve, 2018). Where organizations search for solutions is important because it precedes the selection of a specific behavioral response, and thus the different types of responses and disparate outcomes to performance shortfalls reported in prior research could be explained by the hitherto unexplored locus of search choices deriving from performance feedback to multiple goals (Greve, 2018; Posen et al., 2018).

### Prior Research on the Locus of Search

Research on the locus of search has been scarce (Greve, 2018; Posen et al., 2018) and limited to situations of performance relative to a single goal (Greve, 2018). We, therefore, start our theory building from a review of studies that carry potential implications for how performance feedback on single goals affects the locus of search. Two broad streams exist. One stream of studies, indirectly related to the locus of search, focuses on the type of changes low-performing organizations are more likely to make. To illustrate, Kuusela, Keil, and Maula (2017) show that the likelihood of acquisitions decreases with the magnitude of performance shortfalls, whereas the likelihood of divestments increases. Eggers and Suh (2019) show that the launch of new products in domains with prior experience, or the launch in new domains, depends upon whether performance feedback stems from novel products or products with prior experience. However, the locus of change and the locus of search do not necessarily align since search is an antecedent of change, and not all search results in change. Hence, an organization may search in multiple domains but may choose to change based on the results of only some of these search efforts.

While still conflating search and change, a second stream seems to get closer to the locus of search choices. Noteworthy in this stream is a study by Baum and colleagues (2005) that focuses on where investment banks search for bank partners in underwriting deals. Applying insights from performance feedback theory, they find that decision-makers in an organization performing slightly below aspirations are more inclined to search for bank partners within their existing network rather than outside their network, presumably because they need only small and safer performance improvements anchored in current solutions. In contrast, they find that decision-makers in an organization performing far below its aspiration level are more likely to search for partners beyond their network because they likely need riskier solutions that may provide a greater chance of raising the organization's performance to the aspiration level. Similarly, Iyer, Baù, Chirico, Patel, and Brush (2019) suggest that the larger a performance shortfall, the more likely an organization is to make unrelated rather than related acquisitions. Kavusan and Frankort (2019) found that, with increasing performance shortfalls, firms form new alliances with novel partners yet focus on existing resources. Although this line of work points to the likelihood of a more far-reaching influence of low performance on where search activities occur (e.g., outside an organization's network or to unrelated industries), it still does not examine search in isolation from the decisions that capitalize on search. For example, acquisition decisions build on search that generates candidate firms which a focal organization considers acquiring, but not all are acquired. It is incorrect, therefore, to infer from the decisions ultimately made the locus of search preceding them. Prior work, mentioned above, also falls short of providing a coherent theoretical argument of the locus of search, and how performance feedback—including performance on multiple goals—regulates it.

### Performance on a Primary Goal and the Locus of Search

To derive predictions regarding the implications of performance feedback for the locus of search, it is useful to distinguish between local and distal search (Cyert & March, 1963). Cyert and March (1963) suggested that search may be local along multiple dimensions, specifically close to current activities and within the unit where these activities take place. Research on technological search that has developed largely independently of the performance feedback theory (Posen et al., 2018) has introduced a similar distinction by differentiating between search that occurs along two dimensions: technological and organizational (Rosenkopf & Nerkar, 2001; Rothaermel & Alexandre, 2009). For responses to performance feedback, this implies that low-performing organizations face the choice to search locally or distally along the technological dimension, directing search to areas that build upon familiar or unfamiliar knowledge, and the choice to search locally or distally along the organizational dimension, directing solution search within or outside the organization.

Central to either choice are important differences in the implementation and outcomes of local and distal search (Laursen, 2012; Rosenkopf & Nerkar, 2001). Local search, for instance, is often considered easier to implement, less costly, and less risky (Rosenkopf & Almeida, 2003). For local search along the technological dimension, the key argument is that knowledge and resources are familiar, and thus the organization needs less cognitive effort to assess and coordinate solutions across (local vs. distant) knowledge areas (Knudsen & Levinthal, 2007). This, in turn, may generate better (shortterm) performance (Taylor & Greve, 2006). For local search along the organizational dimension, the key argument goes beyond familiarity and relates to the increased control over the search process and the innovations it generates (Pisano, 1990). However, local search along either dimension may not create sufficient novelty to fix serious problems, constraining the development of truly innovative products (Reed & DeFilippi, 1990) and potentially leading the organization into competency traps (Levitt & March, 1988). In other words, local search—either along the organizational or the technological dimension—is often viewed as less risky but also more limited in the rewards it can provide.

In contrast, distal search allows the focal firm to absorb and integrate knowledge from unfamiliar technological areas or from other organizations (Rosenkopf & Nerkar, 2001), and may therefore lead to more novel solutions and more innovative products that increase sales (Eklund & Kapoor, 2022; Laursen & Salter, 2006; Monteiro & Birkinshaw, 2017). However, organizations engaging in distal search along the organizational dimension may also need to accept the limited control and potentially higher costs it involves, and may need to develop the capacity to effectively absorb external knowledge (Cohen & Levinthal, 1990). Similarly, organizations engaging in distal search along the technological dimension may often need to absorb new knowledge and accept the higher risk of failure and inherent uncertainty related to unfamiliar knowledge areas. In other words, distal search across either dimension may potentially have higher rewards, but these come with an associated increase in risk.

Previous studies suggest that organizations generally prefer local search (Laursen, 2012; Laursen & Salter, 2006; Rosenkopf & Nerkar, 2001). However, following performance feedback theory, we expect that the more performance falls below aspirations on a primary goal, the higher the organization's propensity to perform distal search across both the technological and the organizational dimension. Specifically, the arguments of Baum and colleagues (2005) and Eggers and Suh (2019) imply that, with growing underperformance, organizations will increasingly be willing to take the higher risks associated with distal search. According to Cyert and March (1963) and Greve (2003a, 2003b), these effects are especially likely when increasing performance shortfalls relate to goals of high importance in an organization's goal hierarchy. When organizations underperform on a primary goal, they are more likely to expand their search efforts outside their technological or organizational boundaries to acquire resources and find novel solutions they lack, thus being more willing to tolerate high risks to fix serious problems. Therefore, performance below aspirations on a primary goal leads to an increased propensity to engage in distal search across both the technological and the organizational dimensions. We hypothesize:

*Hypothesis 1:* Performance feedback below aspirations on a primary goal will lead to more distal search along (a) the technological dimension (i.e., unfamiliar technological areas) and (b) the organizational dimension (i.e., external to the organization).

### Performance on Multiple Goals, Self-Enhancement, and the Locus of Search

While Hypothesis 1, consistent with most prior research, considers only the primary goal in setting the locus of search, next, we incorporate performance feedback regarding multiple goals. Our primary thesis is that the self-enhancement motive also plays a central role in regulating the locus of search choices.

Specifically, we suggest that when faced with inconsistent feedback, with low performance on a primary goal and high performance on a secondary goal, decision-makers will direct the locus of search from distal to local search. Local search is aligned with a positive self-image because it draws upon knowledge familiar to the organization and generated inside the organization, whereas distal search is aligned with a negative self-image as familiar knowledge and knowledge generated within the organization does not suffice to address the performance issues faced by the organization. Since situations of feedback inconsistency create ambiguity and room for interpretation (Audia & Brion, 2007), self-enhancing decision-makers may not only favorably assess performance but also choose search directions that protect their self-image while still offering plausible solutions. Building on the ambiguity generated by inconsistent performance signals, decision-makers are able to reprioritize where they search by selecting solution spaces that enhance their image of competence. Directing search within organizational boundaries or in familiar technological areas suggests that the organization and its decision-makers have the necessary knowledge and skills to develop novel solutions. In contrast, searching outside the technological and organizational landscapes suggests that a firm's competence is insufficient to produce good-enough solutions. Furthermore, local search reinforces the credibility of previous choices. It serves as a signal of trust to an organization's personnel. By contrast, distal search can be interpreted as an indication that a firm's efforts and capabilities are not valued, which, in turn, may hurt decision-makers’ self-image and give a negative signal to the market that can jeopardize the competitive position of the organization (Cole & Chandler, 2019; Levinthal & Rerup, 2021). Searching outside the organization, therefore, often faces internal resistance (Antons & Piller, 2015; Laursen & Salter, 2006) and can be perceived as a threat that self-enhancing decision-makers prefer to avoid.

Taken together, under conditions of ambiguity that stem from inconsistent feedback, self-enhancing decision-makers will be inclined to reduce distal search and pursue more local search that signals that the organization has what it takes to fix performance problems. Hence, we hypothesize:

*Hypothesis 2:* Inconsistent performance feedback with performance on a primary goal below aspirations and performance on a secondary goal above aspirations will lead to less distal search along (a) the technological dimension (i.e., unfamiliar technological areas) and (b) the organizational dimension (i.e., external to the organization).

It is important to note that these hypothesized effects of inconsistent feedback from multiple goals in guiding the locus of search are independent of the effects of this feedback in reducing the intensity of problemistic search. While self-enhancement theory suggests that with inconsistent feedback (additional) problemistic search is suppressed and, therefore, the overall intensity of search remains unchanged or is even reduced, we propose that the organization may redirect its (remaining) search behavior towards less distal (more local) landscapes.

## Methods

### Sample and Data

We test our hypotheses in the context of the global pharmaceutical industry. This industry constitutes an appropriate empirical setting to examine our arguments because a hierarchy of (R&D) goals is well documented, performance is regularly monitored at different points of the R&D process, and the search for solutions outside the firm's technological and organizational boundaries plays an important role (Higgins & Rodriguez, 2006; Hoang & Rothaermel, 2010; Macher & Boerner, 2012; Nishimura & Okada, 2014; U.S. Food and Drug Administration, 1999).

Specifically, the pharmaceutical clinical trials process is an excellent context because it allows us to assess different goals within a hierarchy of clinical trials’ goals and alternative responses to examine the locus of search. In the pharmaceutical industry, the goal of innovation performance at the R&D portfolio level can be subdivided into multiple (sub)goals, such as goals related to the number of drug candidates passing the early stages (i.e., Preclinical and Phase I) and the number of drug candidates passing the late stages (i.e., Phases II and III) of the clinical trials process each year. By paying attention to performance feedback regarding such goals, pharmaceutical companies can modify their R&D portfolio choices.

The goals in clinical trials form a clear hierarchy of importance, where the goals of late-stage clinical trials are more important given that performance below aspirations at late stages has a direct impact on innovation performance and financial performance as it means that the firm will have fewer new drugs that it can launch in the market. Further, late-stage trials incur substantially higher costs (Kola & Landis, 2004), and attrition at this stage may create substantial market pressure on pharmaceutical firms (Frantz, 2006). In contrast, early-stage clinical trials’ goals are less important because they have a less direct impact on innovation performance and financial performance. Succeeding in early clinical trials does not impact the near-term potential launch of drugs in the market and, conversely, not meeting early-stage goals is not a fatal failure since firms have plenty of time to make adjustments (Mathieu, 2002; U.S. Food and Drug Administration, 1999).

Research on the R&D process of pharmaceutical firms parallels the hierarchical distinction between early- and late-stage clinical trials and, more specifically, recommends grouping Preclinical and Phase I as early-stage trials because they focus on the safety dimension of drug development, and Phase II and Phase III as late-stage trials because they focus on the efficacy dimension of drug development (Higgins & Rodriguez, 2006; Lee, Lee, Wu, Lee, & Chen, 2005; Nishimura & Okada, 2014).

Our sample consists of 98 global incumbent pharmaceutical firms with a portfolio of clinical trials regulated by the U.S. Food and Drug Administration (FDA) from 1993 until the end of 2021^1,^^2^ (2370 firm-year observations in total), representing more than 80% of sales in the industry, worldwide (as compiled by IMS sales data). We chose to sample global incumbent firms (i.e., firms that were active in the pharmaceutical industry prior to the emergence of biotechnology; for a similar approach please see Grigoriou & Rothaermel, 2017; Hess & Rothaermel, 2011; Rothaermel & Thursby, 2007) because only these companies have the resources to support a portfolio of multiple, high-cost clinical trials over an extended period of time. This allowed us to collect reliable clinical trial data and test our theory across the study period. Further, to account for major mergers and acquisitions that occurred between incumbents in our sample (there were eight such events in total), we followed the prior research and treated these companies as combined entities throughout the entire study period (Nerkar & Roberts, 2004). An additional analysis in which these companies were treated as separate entities until the year of the merger or acquisition produced similar results.

To measure our main dependent and independent variables, we collected clinical trial data from Inteleos, Citeline's Pharmaprojects, and Trialtrove databases. These databases provide extensive information regarding the clinical trials pipeline of the global pharmaceutical industry and are widely utilized to study R&D processes in the management literature (Hess & Rothaermel, 2011; Sosa, 2013). In addition, we collected financial data from Compustat's Annual Updates Fundamentals database, which includes global companies’ annual historical financial data from 1987 onward.

### Dependent Variable

We operationalize distal search along two search dimensions: the technological (i.e., unfamiliar search) and the organizational dimension (i.e., external search). Unfamiliar and external (vs. familiar and internal) search activities represent distal (vs. local) search that reflects critical decisions in R&D portfolio management, capturing the direction and related adjustments of the firm's R&D behavior (e.g., He & Wong, 2004; Lavie, Stettner, & Tushman, 2010; Nishimura & Okada, 2014; Uotila, Maula, Keil, & Zahra, 2009). Our measure of unfamiliar search is the number of drug candidates that enter clinical trials in a focal year in a disease area in which the company did not have clinical trials the previous 5 years. To create this measure, we relied on the list of the 14 main disease areas provided by Citeline's Pharmaprojects (please see the Appendix in the online supplemental material). Our measure of external search is the number of drug candidates entering clinical trials in a focal year that were acquired through licensing or acquisitions. To identify whether drug candidates were externally sourced by our sampled firms, we used related information provided by Inteleos and Citeline's Pharmaprojects databases. In a series of posthoc tests, we also use as our dependent variable ratios obtained by dividing the abovementioned variables by the total number of drug candidates in clinical trials initiated during the focal year. We report these models in the additional analyses section following the results of our main analyses.

### Independent Variables

We compute two performance measures following four steps. First, we calculated performance on a primary goal as the number of drug candidates passing the late stages (i.e., Phases II and III) of the clinical trials process, and performance on a secondary goal as the number of drug candidates passing the early stages (i.e., Preclinical and Phase I). These provide concrete evidence regarding the efficacy (primary goal) and safety (secondary goal) of the drug candidate portfolio and are regularly monitored by pharmaceutical firms during their drug development process (Mathieu, 2002; U.S. Food and Drug Administration, 1999).

Second, we created a measure for the performance aspiration level (Bromiley & Harris, 2014; Greve, 1998). Following Cyert and March (1963) and more recent work (Blettner, He, Hu, & Bettis, 2015; Greve, 2003a; O’Brien & David, 2014), we use a combination of the firm's social and historical early- and late-stage aspirations to calculate performance aspiration level^3,^^4^ based on the assumption that pharmaceutical companies concurrently evaluate (a) whether they are performing better or worse than their competitors at gaining the approval of regulatory bodies (Schulze & Ringel, 2013; i.e., social aspirations), and (b) whether their performance constitutes an improvement or a deterioration in relation to their own past outcomes (Hess & Rothaermel, 2011; i.e., historical aspirations). Social (SA), historical (HA), and overall (AL) aspirations levels were calculated using the following formulas, where *t* is the time; *i* refers to the focal firm; *p* refers to the early (Preclinical and Phase I) or late (Phase II and Phase III) stages of clinical trials; *N* is the total number of firms (including *i*) conducting clinical trials in the same disease area as focal firm *i*; *j* refers to each of the *N* competing firms, excluding focal firm *i* (i.e., *j* ≠ *i*); and a_1_ and a_2_ are the corresponding weights for historical and social aspirations, respectively.

$$SA_{ipt}=\frac{\sum_{\;j\neq i\;}\;Successful\;exits\;from\;clinical\;trials\;phases_{\;jt}}{N-1}$$

$$HA_{ipt}=\;a_{1}HA_{ip(t-1)}+(1-a_{1})Performance_{ip(t-1)}$$

$$AL_{ipt}=\;a_{2}SA_{ipt}+(1-a_{2})HA_{ipt}$$

Following Greve's (2003a) approach, we empirically determined the values for a_1_ and a_2_ that best fit the data. This procedure yielded a value of 0.8 for a_1_ and a value of 0.9 for a_2_. These values suggest that, in this particular context, (a) pharmaceutical firms due to the long process of new drug development assess their performance based on a longer period historical aspiration level (Muttenthaler, King, Adams, & Alewood, 2021) and (b) overall aspirations are mostly driven by social aspirations. This is consistent with the view that in the pharmaceutical sector companies are always racing against their competitors to launch new drugs on the market and, therefore, can be expected to closely monitor their innovation output (Schulze & Ringel, 2013).

Third, we take the absolute value of the firm's actual performance in the early and late stages of clinical trials minus the aspiration level to capture the distance of firm performance from the aspiration level.

Fourth, following the approach introduced by Greve (1998) to examine theoretically derived discontinuities in the influence of performance across the performance range, we utilize a spline specification. Greve included two categories that together cover the entire range of performance: performance above the aspiration level, and performance below the aspiration level. Examination of the sign and significance of the coefficients of these two variables reveals linearity or discontinuity in the influence of performance on the outcome of interest. The knot or point at which the variable is split is where performance equals the aspiration level, a point that is theoretically derived. We use this approach, which is now widely used in research on the relationship between past performance and change (e.g., Audia & Greve, 2006; Gaba & Greve, 2019), to test Hypothesis 1. We then expand this approach to test Hypothesis 2 by creating four spline variables that together cover the four combinations of performance on two goals. Again, differences in the sign and significance of the coefficients are indicative of non linearity. The two categories concerning performance on a primary goal below the aspiration allow us to test Hypothesis 2. The other two allow us to cover the entire range of performance influences—that is, when performance on a primary goal is above the aspiration.

Specifically, to test Hypothesis 1, we include in the models two distinct variables: one captures situations in which performance on the primary goal is below the aspiration level and is zero when performance is above the aspiration level; and the second captures situations in which performance on the primary goal is above the aspiration level and is zero when performance is below the aspiration level. A positive coefficient of performance below the aspiration level would support Hypothesis 1. We do not formulate a prediction regarding the influence of performance above the aspiration level.

To test Hypothesis 2, we create two measures of performance on multiple goals: consistent negative feedback and inconsistent feedback with the primary goal below the aspiration level. Consistent negative feedback captures situations in which both performance on the primary goal and performance on the secondary goal are below the aspiration level. This measure is equal to the absolute value of the difference between performance and overall aspiration level for early-stage clinical trials (secondary goal) when performance is below the aspiration level multiplied by the absolute value of the difference between performance and aspiration level for late-stage clinical trials (primary goal) when performance is below aspirations. This variable takes the value of zero for other combinations of feedback on the primary and secondary goal (i.e., when the performance in at least one of these two goals exceeds aspirations).

Inconsistent feedback captures situations in which performance on the primary goal is below the aspiration level and performance on the secondary goal is above the aspiration level. When this inconsistency occurs, this variable equals the absolute value of the difference between performance and overall aspiration level for late-stage clinical trials multiplied by the value of the difference between performance and overall aspiration level for early-stage clinical trials. This variable equals zero in other combinations of feedback on late- and early-stage clinical trials. A negative and significant coefficient for inconsistent feedback would lend support to Hypothesis 2.

To cover the entire range of performance situations when both goals are considered, we also include in the analyses a measure of consistent positive feedback when performance is above the aspiration level for both goals and a measure of inconsistent feedback in which performance on the primary goal is above the aspiration level and performance on the secondary goal is below the aspiration level. We do not formulate predictions about the influence of these two performance situations, although we report their implication for the overall pattern of findings.

### Control Variables

We control for the number of patented molecules (i.e., patents that include at least one Derwent compound number) that the focal company registered in the last 10 years in any patent office included in the Derwent database. The availability of a larger number of patented molecules may influence whether firms choose to search distally or locally when initiating clinical trial projects. Because this variable is positively skewed and has many zero values, we use the natural logarithm of the number of patented molecules. We also control for the total number of licensing-in opportunities by including a variable that captures the number of R&D alliances and acquisitions that the company formed during the focal and the last 4 years. We used Refinitiv's SDC database to identify the R&D alliances and acquisitions formed by our sampled pharmaceutical firms.

We control for firm size, which we measure as the natural logarithm of the number of employees in a firm. We also control for firm-level financial performance aspirations, which we measure using return on total assets (ROA) and operationalize in a similar manner as our main independent variables (e.g., Greve, 2003a). To control for market-level factors, we include the competitive intensity in the R&D market measured as the natural logarithm of the number of competing clinical trial projects in each disease area in a given year. Finally, to control for time and origin effects, we include year and country (the United States, the European Union, and the rest of the world) dummies, respectively.

The theory of performance feedback views performance on goals as providing information that influences the choices that organizations make, but performance also leads to an accumulation or a depletion of resources. To account for the possibility that the analysis of the influence of performance reflects potential “resource” effects, we control for several indicators of resources. We reason that any “resource” effect will be reflected in the coefficients of these variables. First, we control for R&D capability operationalized as a firm's annual investment in R&D divided by its sales for that year. Second, we control for three different types of slack resources: (a) unabsorbed slack—the ratio of cash and marketable securities to liabilities; (b) absorbed slack—the ratio of selling, general, and administrative expenses to sales; and (c) potential slack—the ratio of debt to equity (e.g., Greve, 2003a). In addition, because a firm's age is often seen as a proxy for the availability of resources to innovate (Kotha, Zheng, & George, 2011), we control for firm age as the number of years since incorporation.

Finally, while our main interest lies in examining situations in which performance on a primary goal is below the aspiration level, as we mention in our description of the computation of the independent variables, we also include measures that capture situations in which performance on the primary goal is above the aspiration level.

## Results

Our data is structured as a panel dataset with the firm-year period as the unit of analysis and multiple observations for each firm. Because the dependent variables are counts that show overdispersion (chisquare for alpha equal zero = 515.26 for unfamiliar search; chisquare for alpha equal zero = 29.95 for external search), we employ a negative binomial regression estimator. Following Allison and Waterman (2002), we estimate fixed effects by including a dummy variable for each firm. In the Additional Analyses section, we report results obtained using OLS regression with fixed effects in which the dependent variables are ratio measures of the dependent variables.

Table 1 presents descriptive statistics, whereas Table 2 presents the pairwise correlations of our variables. Table 3 reports results for unfamiliar search—the number of drug candidates that enter clinical trials in a focal year in a disease area in which the company did not have clinical trials in the previous 5 years—whereas Table 4 reports results for external search—the number of drug candidates entering clinical trials in a focal year that were acquired through licensing or acquisitions. In Table 1, apart from the expected high correlations among some of the performance variables (e.g., consistent negative feedback and performance feedback with primary goal below), all bivariate correlations are below the commonly used threshold of 0.60, thereby mitigating concerns about multicollinearity. Variance inflation factors (VIFs) for the coefficients are also well below the most commonly used cut-off values as the maximum VIF is 2.12 (Cohen, Cohen, West, & Aiken, 2013), providing further evidence that multicollinearity is not a problem in our analyses.

**Table 1:** Descriptive Statistics

Variable	*M*	*SD*	Min	Max
1. Unfamiliar search	0.97	2.02	0.00	8.00
2. External search	0.36	2.25	0.00	19.00
3. Performance feedback with primary goal below	1.42	2.62	0.00	8.72
4. Inconsistent feedback with primary goal below	0.24	1.17	0.00	10.21
5. Consistent negative feedback	4.12	10.35	0.00	42.72
6. Number of employees (log)	3.96	0.34	3.77	5.02
7. Performance feedback ROA below	0.03	0.05	0.00	0.80
8. Performance feedback ROA above	0.03	0.05	0.00	0.86
9. Number of clinical trial projects active (log)	5.80	0.95	0.69	6.77
10. R&D intensity	0.22	0.15	0.00	0.50
11. Unabsorbed slack	446.84	1,955.04	0.00	24,542.01
12. Absorbed slack	0.78	16.16	0.00	568.00
13. Potential slack	0.22	0.72	0.00	25.11
14. Firm age	34.90	7.81	18.00	84.00
15. Number of alliances initiated the last 5 years	4.18	9.72	0.00	61.00
16. U.S. headquartered	0.11	0.31	0.00	1.00
17. E.U. headquartered	0.20	0.40	0.00	1.00
18. Number of patented molecules (log)	3.28	1.84	0.69	10.29
19. Performance feedback with primary goal above	1.36	4.14	0.00	18.75
20. Inconsistent feedback with primary goal above	0.60	2.54	0.00	12.56
21. Consistent positive feedback	56.37	311.75	0.00	4,138.23

**Table 2:** Correlation Matrix

Variable	1	2	3	4	5	6	7	8	9	10	11	12	13	14	15	16	17	18	19	20
1. Unfamiliar search																				
2. External search	0.33																			
3. Performance feedback with primary goal below	0.47	0.35																		
4. Inconsistent feedback with primary goal below	−0.07	−0.01	0.36																	
5. Consistent negative feedback	0.39	0.17	0.83	−0.08																
6. Number of employees (log)	0.06	−0.05	0.01	0.04	−0.03															
7. Performance feedback ROA below	0.02	0.04	0.05	−0.02	0.06	−0.05														
8. Performance feedback ROA above	−0.10	−0.02	−0.08	0.10	−0.09	0.03	−0.24													
9. Number of clinical trial projects active (log)	0.01	0.02	0.05	0.09	0.01	0.02	0.00	0.02												
10. R&D intensity	−0.16	0.01	−0.07	0.00	−0.03	−0.26	0.02	0.10	0.02											
11. Unabsorbed slack	−0.08	−0.03	−0.06	0.01	−0.08	0.20	−0.07	0.13	0.01	−0.12										
12. Absorbed slack	−0.01	−0.01	−0.02	−0.01	−0.01	−0.02	0.01	−0.02	−0.04	−0.03	−0.01									
13. Potential slack	−0.05	0.00	−0.03	−0.02	−0.02	−0.04	0.18	−0.04	0.02	0.09	0.05	−0.01								
14. Firm age	−0.02	−0.01	−0.07	0.03	−0.11	−0.04	−0.02	0.03	−0.02	0.16	0.02	0.01	−0.01							
15. Number of alliances initiated the last 5 years	0.08	0.34	0.08	0.08	−0.06	0.22	0.00	−0.02	0.02	−0.28	0.15	−0.01	−0.02	−0.08						
16. U.S. headquartered	−0.06	−0.04	−0.03	0.02	−0.04	0.13	−0.03	0.24	0.02	−0.14	0.59	−0.01	0.09	−0.13	0.26					
17. E.U. headquartered	−0.06	−0.02	−0.09	−0.07	−0.06	0.01	−0.03	−0.07	0.00	0.21	−0.11	−0.02	−0.02	−0.01	−0.10	−0.17				
18. Number of patented molecules (log)	0.08	0.06	0.03	0.10	−0.04	0.20	−0.11	0.01	0.03	−0.16	0.23	0.02	−0.05	−0.08	0.33	0.20	0.05			
19. Performance feedback with primary goal above	−0.09	−0.04	0.45	0.06	0.56	0.08	0.02	−0.05	0.00	−0.05	0.32	−0.01	−0.01	−0.16	0.23	0.16	−0.06	0.10		
20. Inconsistent feedback with primary goal above	0.01	−0.01	−0.06	−0.05	−0.09	0.02	−0.02	−0.02	−0.01	−0.07	0.02	0.11	−0.04	−0.07	0.03	−0.04	0.04	0.07	−0.02	
21. Consistent positive feedback	−0.04	−0.03	−0.09	−0.04	−0.07	0.19	0.01	−0.03	0.00	−0.12	0.18	−0.01	−0.03	0.06	0.38	0.14	−0.09	0.20	0.74	−0.04

*Note:* All absolute correlation values above 0.05 are significant in *p* < .05. ROA = return on assets; R&D = research and development.

**Table 3:** Negative Binomial Regression Analysis of Unfamiliar Search With Year and Company Dummies

	Unfamiliar Search								
	M1			M2			M3		
	β	*SE*	*P*	β	*SE*	*P*	β	*SE*	*P*
Performance feedback with primary goal below				0.31	0.03	.000			
Inconsistent feedback with primary goal below							−0.38	0.07	.000
Consistent negative feedback							0.08	0.01	.000
Number of employees (log)	−0.60	0.25	.015	−0.28	0.23	.226	−0.40	0.21	.064
Performance feedback ROA below	−1.35	1.66	.419	−1.99	1.53	.195	−1.89	1.50	.206
Performance feedback ROA above	−0.59	1.79	.742	−1.85	1.63	.256	−0.91	1.91	.635
Number of clinical trial projects active (log)	−0.05	0.06	.455	−0.07	0.06	.201	−0.03	0.05	.571
R&D intensity	−0.23	0.61	.703	−0.40	0.56	.482	0.24	0.53	.654
Unabsorbed slack	0.00	0.00	.000	0.00	0.00	.000	0.00	0.00	.000
Absorbed slack	−0.01	0.02	.656	−0.01	0.02	.615	−0.01	0.02	.599
Potential slack	−0.05	0.23	.833	0.10	0.21	.621	−0.15	0.21	.471
Firm age	−7.34	0.07	.000	−6.75	0.06	.000	−6.42	0.06	.000
Number of alliances initiated the last 5 years	0.01	0.01	.225	−0.01	0.01	.054	0.01	0.01	.039
U.S. headquartered	0.50	0.92	.589	0.81	0.83	.324	0.31	0.76	.682
E.U. headquartered	0.43	0.78	.577	0.55	0.70	.426	0.50	0.67	.451
Number of patented molecules (log)	0.03	0.07	.689	0.05	0.07	.435	−0.02	0.06	.782
Performance feedback with primary goal above				−0.04	0.04	.254			
Inconsistent feedback with primary goal above							−0.01	0.02	.502
Consistent positive feedback							0.00	0.00	.723
Constant	168.27	0.00	.000	153.55	0.00	.000	146.48	0.00	.000
Observations	2,370			2,370			2,370		
Log likelihood	−2,141.11			−2,067.71			−2,022.91		

**Table 4:** Negative Binomial Regression Analysis of External Search With Year and Company Dummies

	External Search								
	M4			M5			M6		
	β	*SE*	*P*	β	*SE*	*P*	β	*SE*	*P*
Performance feedback with primary goal below				0.22	0.09	.015			
Inconsistent feedback with primary goal below							−0.37	0.19	.049
Consistent negative feedback							0.02	0.03	.452
Number of employees (log)	−4.76	1.42	.001	−4.17	1.39	.003	−4.87	1.47	.001
Performance feedback ROA below	−2.76	6.68	.680	−2.40	6.31	.703	−2.62	6.47	.685
Performance feedback ROA above	2.75	7.00	.695	0.16	7.23	.982	1.20	7.98	.881
Number of clinical trial projects active (log)	−0.03	0.25	.915	−0.13	0.24	.591	−0.07	0.24	.764
R&D intensity	−0.61	2.29	.789	−0.90	2.20	.684	0.17	2.25	.940
Unabsorbed slack	0.00	0.00	.258	0.00	0.00	.989	0.00	0.00	.380
Absorbed slack	0.05	1.83	.979	0.10	1.92	.961	1.21	1.81	.503
Potential slack	0.87	1.10	.427	0.37	0.98	.703	0.47	1.04	.651
Firm age	−4.52	1.80	.012	−0.07	486.21	1.000	−1.37	158.70	.993
Number of alliances initiated the last 5 years	0.06	0.02	.000	0.05	0.02	.007	0.06	0.02	.001
U.S. headquartered	6.29	40.97	.878	11.86	947.97	.990	9.51	240.68	.968
E.U. headquartered	5.35	41.00	.896	−0.73	1,776.66	1.000	0.77	323.48	.998
Number of patented molecules (log)	−0.06	0.32	.854	−0.07	0.30	.823	−0.08	0.31	.790
Performance feedback with primary goal above				−0.79	0.37	.030			
Inconsistent feedback with secondary goal below							−0.24	0.10	.018
Consistent positive feedback							−0.01	0.00	.106
Constant	114.44	0.00	.000	4.87	12,108.81	1.000	39.29	3,835.75	.992
Observations	2,370			2,370			2,370		
Log likelihood	−558.69			−549.68			−551.65		

Focusing first on the control variables, Model 1 in Table 3 indicates that firms that are older, firms with greater unabsorbed slack and, to a lesser, extent firms with more employees, are all less likely to engage in unfamiliar search. Model 4 in Table 4 points to a different but sensible pattern for the control variables where firms with a larger number of alliances in the last 5 years engage in more external search and firms with U.S. headquarters engage in less external search.

Turning to our first hypothesis, we first observe in Model 2 in Table 3 a positive and significant coefficient (β = 0.31; *p* = .000). This indicates that the more performance falls below aspirations on a primary goal, the higher the organization's propensity to engage in unfamiliar search. Using the margin command in Stata, we find that when performance on the primary goal drops by a standard deviation, the unfamiliar search increases by approximately 0.8 more unfamiliar projects. Similarly, in Model 5 in Table 4 we find that the coefficient for performance feedback below aspirations on a primary goal is positive and significant (β = 0.22; *p* = .015), indicating that the more performance on a primary goal falls below aspirations, the higher is the organization's propensity to engage in external search. In this case, when performance on the primary goal drops by a standard deviation, the external search increases by approximately 0.3. Taken together, these results provide support for Hypothesis 1. Note that Models 2 (Table 3) and 5 (Table 4) also include the variable performance feedback with primary goal above aspirations. The coefficient for this variable is negative, although only significant for external search (β = −0.04; *p* = .254; Model 2, Table 3; β = −0.79; *p* = .030; Model 5, Table 4). These results are consistent with a tendency for high-performing firms to reduce the propensity to engage in distal search on both the technological and the organizational dimension.

To test Hypothesis 2, in Model 3 (Table 3) and Model 6 (Table 4) we add to consideration performance on the secondary goal. Specifically, we drop the single goal measure of performance with primary goal below and replace it with two variables: inconsistent feedback with primary goal below the aspiration level and consistent negative feeback. Model 3 in Table 3 shows that the coefficient for the inconsistent feedback variable is negative and significant (β = −0.38; *p* = .000) whereas the coefficient for the consistent negative feedback variable is positive and significant (β = 0.08; *p* = .000). Specifically, when performance on a primary goal drops by a standard deviation and performance on a secondary goal is high—that is, one standard deviation above the mean value of performance feedback from the secondary goal above—unfamiliar search decreases by approximately 0.6 unfamiliar projects. Conversely, when performance on a primary goal drops by a standard deviation and performance on a secondary goal is low—that is, one standard deviation below the mean—unfamiliar search increases by approximately 0.8 unfamiliar projects. Moving to Model 6 in Table 4, we find a negative and significant coefficient for the inconsistent feedback variable (β = −0.37; *p* = .049; Model 3, Table 4). Here, however, the coefficient for the consistent negative feedback is positive but not significant (β = 0.02; *p* = .452). The negative and significant coefficient for the inconsistent feedback variable implies that when performance on a primary goal drops by a standard deviation and performance on a secondary goal is high—that is, one standard deviation above the mean value of performance feedback from secondary goal above—external search decreases by approximately 1.2 external projects. Overall, the results in Model 3 in Tables 3 and Model 6 in Table 4 provide strong support for Hypothesis 2 which holds that in cases of inconsistent feedback, where performance on a primary goal is below aspirations but, at the same time, performance on a secondary goal is above aspirations, decision-makers’ propensity to engage in distal (unfamiliar or external) search decreases.

While the main focus here is on situations in which performance on a primary goal falls below the aspiration, to provide a complete account of the influence of performance on the propensity to engage in distal search, we considered also the influence of situations in which performance on the primary goal is above the aspiration. Model 3 in Table 3 shows negative but nonsignificant coefficients for the inconsistent feedback variable (performance on primary goal above aspiration but performance on secondary goal below aspiration) and for the consistent positive feedback variable (performance on primary goal above aspiration and performance on secondary goal above aspiration). These coefficients mirror the negative but not significant coefficient in Model 2, Table 3. Model 6 in Table 4 shows negative cofficients for both variables significant at least at the .10 level. These results therefore confirm, as a general yet weak trend, a propensity by firms in our sample to reduce distal search when performance on the primary goal is above the aspiration level, regardless of whether performance on a secondary goal is above or below the aspiration level. Our theoretical analysis is consistent with this pattern because we have suggested that the tendency to shy away from distal search is strengthened by the perception of threat that is felt when performance on a primary goal falls below the aspiration level. Lastly, we run models replacing the spline variables with the interaction between performance on primary goal below aspirations and performance on secondary goal above aspirations. While the negative binomial regression does not converge, we are able to report the results obtained using OLS regression, in which which we use the ratio variables. While the interaction does not allow to estimate separate coefficients for situations of consistent and inconsistent feedback, the result is in line with our prediction as the interaction term shows a negative and significant coefficient on both unfamiliar search and external search (β = −0.03; *p* = .000 for unfamiliar search, and β = −0.21; *p* = .000 for external search).

### Additional Analyses

#### Model with the intensity of search

It is helpful to consider the influence of performance on the total number of drug candidates entering clinical trials (i.e., total count of search), irrespective of whether they are in unfamiliar disease areas or external to the organization. This dependent variable approximates the intensity of search rather than its locus. We report the results in Table 5. We observe that search intensity goes up when performance on the primary goal is below the aspiration level (β = 0.27; *p* = .000; Model 8, Table 5). We also observe that this effect remains for consistent negative feedback (β = 0.08; *p* = .000; Model 8, Table 5) but disappears for inconsistent feedback (β = 0.04; *p* = .205; Model 8, Table 5). An explanation of this pattern is that self-enhancing assessments of performance turn off the impetus for more search (Jordan & Audia, 2012). Considered in conjunction with the results of our main analyses, the pattern emerging in our sample is that when performance on a primary goal is below the aspiration level and performance on a secondary goal is above the aspiration level, not only does the intensity of search not increase—as predicted by research that focuses on single goals—but distal search decreases.

**Table 5:** Negative Binomial Regression Analysis of Total Search With Year and Company Dummies

	Total Search								
	M7			M8			M9		
	β	*SE*	*P*	β	*SE*	*P*	β	*SE*	*P*
Performance feedback with primary goal below				0.27	0.02	.000			
Inconsistent feedback with primary goal below							0.04	0.03	.205
Consistent negative feedback							0.08	0.01	.000
Number of employees (log)	−0.37	0.20	.067	−0.14	0.19	.460	−0.33	0.18	.076
Performance feedback ROA below	−0.68	1.47	.645	−1.48	1.36	.276	−1.60	1.37	.244
Performance feedback ROA above	−0.75	1.40	.589	−1.31	1.32	.321	−1.13	1.27	.371
Number of clinical trial projects active (log)	−0.04	0.05	.456	−0.07	0.05	.138	−0.04	0.05	.383
R&D intensity	0.44	0.50	.376	0.44	0.46	.345	0.60	0.46	.193
Unabsorbed slack	0.00	0.00	.007	0.00	0.00	.024	0.00	0.00	.119
Absorbed slack	−0.07	0.38	.847	−0.01	0.02	.670	−0.01	0.03	.731
Potential slack	−0.03	0.12	.824	−0.03	0.11	.804	−0.04	0.12	.739
Firm age	−0.18	0.46	.698	0.01	0.42	.977	−0.13	0.42	.763
Number of alliances initiated the last 5 years	0.02	0.00	.000	0.01	0.01	.318	0.03	0.00	.000
U.S. headquartered	0.07	0.74	.924	0.47	0.66	.479	0.21	0.65	.749
E.U. headquartered	0.30	0.72	.675	0.39	0.66	.552	0.39	0.66	.549
Number of patented molecules (log)	0.06	0.06	.351	0.08	0.06	.189	0.01	0.06	.802
Performance feedback with primary goal above				0.05	0.03	.052			
Inconsistent feedback with primary goal above							0.00	0.02	.871
Consistent positive feedback							0.00	0.00	.008
Constant	4.23	11.37	.710	−0.94	10.40	.928	3.10	10.33	.764
									
Observations	2,370			2,370			2,370		
Log likelihood	−2,979.29			−2,901.59			−2,894.19		

#### Ratio measures of the dependent variables

To test the robustness of the results concerning unfamiliar and external search, we ran OLS regression with firm fixed effects and ratio variables as dependent variables (Tables 6 and 7). Unfamiliar search ratio is the number of drug candidates that enter clinical trials in a focal year in a disease area in which the company did not have clinical trials in the previous 5 years divided by the total number of drug candidates in clinical trials initiated during the focal year. External search ratio is the number of drug candidates entering clinical trials in a focal year that were acquired through licensing or acquisitions divided by the total number of drug candidates in clinical trials initiated during the focal year. The pattern of the results emerging from these additional analyses is reassuring. For example, in Tables 6 and 7, Hypothesis 1 (both parts) was confirmed as performance on a primary goal below aspiration has a positive and statistically significant coefficient for both ratio variables (β = 0.02; *p* = .000 in Model 11, Table 6; β = 0.03; *p* = .000 in Model 14, Table 7). Hypothesis 2 was also supported as the inconsistent performance feedback with performance on a primary goal below aspirations and performance on a secondary goal above aspirations has a negative and significant coefficient on the percentage change towards both unfamiliar and external search (β = −0.01; *p* = .000 in Model 12, Table 6; β = −0.01; *p* = .000 in Model 15, Table 7). Preliminary analyses showed the same pattern when we used Poisson regressions with the count dependent variables.

**Table 6:** Panel Data OLS Regression of Unfamiliar Search Ratio With Fixed Effects

	Unfamiliar Search Ratio								
	M10			M11			M12		
	β	*SE*	*p*	β	*SE*	*p*	β	*SE*	*P*
Performance feedback with primary goal below				0.02	0.00	.000			
Inconsistent feedback with primary goal below							−0.01	0.00	.000
Consistent negative feedback							0.00	0.00	.000
Number of employees (log)	−0.03	0.01	.006	−0.03	0.01	.002	−0.03	0.01	.001
Performance feedback ROA below	0.03	0.05	.549	0.01	0.05	.790	0.03	0.05	.618
Performance feedback ROA above	0.04	0.06	.464	0.03	0.06	.556	0.10	0.06	.066
Number of clinical trial projects active (log)	0.00	0.00	.968	0.00	0.00	.995	0.00	0.00	.762
R&D intensity	−0.01	0.02	.684	−0.01	0.02	.719	0.00	0.02	.995
Unabsorbed slack	0.00	0.00	.001	0.00	0.00	.000	0.00	0.00	.016
Absorbed slack	0.00	0.00	.431	0.00	0.00	.421	0.00	0.00	.416
Potential slack	0.00	0.00	.787	0.00	0.00	.737	0.00	0.00	.642
Firm age	0.00	0.00	.047	0.00	0.00	.016	0.00	0.00	.017
Number of alliances initiated the last 5 years	0.01	0.00	.000	0.01	0.00	.000	0.01	0.00	.000
U.S. headquartered	−0.19	0.04	.000	−0.18	0.04	.000	−0.18	0.04	.000
E.U. headquartered	0.01	0.04	.875	0.01	0.04	.871	0.00	0.04	.975
Number of patented molecules (log)	−0.01	0.00	.082	−0.01	0.00	.096	−0.01	0.00	.036
Performance feedback with primary goal above	−0.01	0.00	.000	−0.01	0.00	.000			
Inconsistent feedback with secondary goal below							0.00	0.00	.053
Consistent positive feedback							0.00	0.00	.000
Constant	0.20	0.05	.000	0.21	0.05	.000	0.22	0.05	.000
									
Observations	2,370			2,370			2,370		
*R*-squared	0.30			0.34			0.30		
Number of companies	98			98			98		

**Table 7:** Panel Data OLS Regression Analysis of External Search Ratio With Fixed Effects

	External Search Ratio								
	M13			M14			M15		
	β	*SE*	*p*	β	*SE*	*p*	β	*SE*	*p*
Performance feedback with primary goal below				0.03	0.00	.000			
Inconsistent feedback with primary goal below							−0.01	0.00	.000
Consistent negative feedback							0.01	0.00	.000
Number of employees (log)	0.00	0.01	.801	0.00	0.01	.910	0.00	0.01	.856
Performance feedback ROA below	−0.07	0.07	.308	−0.10	0.06	.088	−0.11	0.06	.067
Performance feedback ROA above	−0.06	0.07	.432	−0.07	0.07	.255	0.01	0.06	.874
Number of clinical trial projects active (log)	0.00	0.00	.951	0.00	0.00	.899	0.00	0.00	.662
R&D intensity	−0.01	0.03	.687	−0.01	0.02	.735	0.02	0.02	.502
Unabsorbed slack	0.00	0.00	.134	0.00	0.00	.517	0.00	0.00	.221
Absorbed slack	0.00	0.00	.693	0.00	0.00	.657	0.00	0.00	.650
Potential slack	0.00	0.00	.438	0.00	0.00	.452	0.00	0.00	.550
Firm age	0.00	0.00	.805	0.00	0.00	.736	0.00	0.00	.576
Number of alliances initiated the last 5 years	0.00	0.00	.000	0.00	0.00	.001	0.00	0.00	.000
U.S. headquartered	−0.09	0.05	.078	−0.08	0.05	.087	−0.09	0.04	.041
E.U. headquartered	0.09	0.05	.114	0.09	0.05	.085	0.08	0.05	.107
Number of patented molecules (log)	0.00	0.00	.866	0.00	0.00	.682	0.00	0.00	.506
Performance feedback with primary goal above	−0.01	0.00	.000	−0.01	0.00	.000			
Inconsistent feedback with primary goal above							0.00	0.00	.665
Consistent positive feedback							0.00	0.00	.078
Constant	0.00	0.07	.966	0.03	0.06	.667	0.02	0.06	.720
									
Observations	2,370			2,370			2,370		
*R*-squared	0.08			0.23			0.28		
Number of companies	98			98			98		

#### Additional robustness and endogeneity tests

First, we tested the sensitivity of our findings to outliers (influential observations) by winsorizing at the 99th percentile variables that have a maximum value higher than the mean plus three standard deviations (as presented in Table 2). The results with these winsorized variables are largely similar to our original findings (for unfamiliar search, Hypothesis 1: β = 0.34, *p* = .000, and Hypothesis 2: β = −0.41, *p* = .012; for external search, Hypothesis 1: β = 0.30, *p* = .032, and Hypothesis 2: β = −0.42, *p* = .044), thus indicating that outliers do not substantially affect our results.

Furthermore, we considered potential endogeneity concerns regarding our two independent variables. Since projects need to pass the early stages of clinical trials to enter the late stages, it is conceivable that performance of projects at late-stage trials may be endogenous to the performance of projects at the early stages at a prior point in time. We believe that the likelihood of this is relatively small because the success factor in each stage is different. Specifically, in the early stages, the success factor is the level of toxicity of the drug, whereas in the late stages, the success factor is the efficacy of the drug compared to other drugs in the market or the full recovery after treatment rate. There is no evidence, for example, that a drug that has successfully finished Phase I as non-toxic will necessarily also be effective in treating a disease. Also, efficacy does not imply safety, as several cases show that promising drug candidates with good efficacy results in late-stage trials eventually need to be terminated because they unexpectedly exhibit safety issues that only get noticed at these later stages. Nonetheless, we ran negative binomial regressions that controlled for the actual number of drug candidates at the early stages and the number of drug candidates at the late stages. Reassuringly, the effects of our independent variables remain largely unaffected (for unfamiliar search, Hypothesis 1: β = 0.35, *p* = .000, and Hypothesis 2: β = −0.46, *p* = .000; for external search, Hypothesis 1: β = 0.32, *p* = .000, and Hypothesis 2: β = −0.14, *p* = .000).

## Discussion

This study offers novel theory and findings regarding the relationship between performance feedback on multiple goals and the locus of search by differentiating between local and distal search across two dimensions. Our results show that, taken by itself, low performance on a primary goal is related to an increased focus on distal search in both the technological and organizational dimensions, but this relationship changes when a secondary goal is taken into consideration. When performance on a primary goal is low, and performance on a secondary goal is high, the focus on distal search declines, indicating that organizations put more effort into finding solutions that draw on internal resources and technologies familiar to their previous activities. This evidence that high performance on a secondary goal alters the direction of responses to low performance on a primary goal extends research on self-enhancement and learning from performance feedback (Audia & Greve, 2021; Jordan & Audia, 2012). Indeed, to our knowledge, this is the first study that proposes and shows a link between situations of performance ambiguity that foster self-enhancement and the choice of the locus of search.

### Theoretical Implications

Our arguments and results add to several streams of research on firm responses to performance feedback. We contribute to studies that examine the relationship between performance feedback on multiple goals and search. Our arguments and results align with and extend the multiple-goal model recently proposed by Audia and Greve (2021), which suggests that considering multiple goals is central to a broader explanation of how organizations utilize performance feedback to regulate behavior. Some related research has recently started to examine behavioral responses to multiple goals (e.g., Gaba & Greve, 2019; Hu & Bettis, 2018; Mazzelli et al., 2019), but it has remained silent on whether and how performance feedback influences the locus of search choices. Our study suggests that multiple goals are of particular importance in explaining the locus of search and, therefore, future research on search should take into account performance feedback regarding constellations of goals.

More specifically, our study relates to and extends research that has examined the effects of inconsistent performance feedback on search. While inconsistent feedback may arise from multiple goals, it may also be the result of different performance referents (e.g., social vs. historical aspirations) or different reference points (e.g., aspirations vs. survival points). Prior research has mainly suggested that such inconsistent performance feedback affects search intensity (Blagoeva et al., 2020; Kostopoulos et al., 2023; Lucas et al., 2018). Our theory and results complement these studies by highlighting a second equally important effect on the locus of search that calls attention to the value of looking beyond search intensity to understand the complex effects of inconsistent feedback.

Building further on this line of reasoning, our study adds to our understanding of the specific locus of search choices. In particular, by theorizing and testing different (local vs. distal) loci of search choices we show that performance feedback has a more complex and more nuanced relationship with search than previously thought. Importantly, focusing on performance on a single (primary) goal alone—as most prior work linking performance feedback to locus of search has done—would leave an important source of heterogeneity regarding locus of search hidden. Our study shows that an understanding of the link between performance and locus of search benefits from considering the constellation of performance feedback on multiple goals because organizations may search locally or distally depending upon different constellations.

Zooming into the different dimensions of the locus of search, our results add new empirical insights to performance feedback theory. While Cyert and March's (1963) discussion of the locus of search introduced multiple dimensions (e.g., locus relative to existing activities and therefore related knowledge and technologies, and locus relative to the unit where the problem occurs), later research has mostly focused on the idea of familiarity either in terms of familiar versus unfamiliar external partners or related or unrelated acquisitions (Baum et al., 2005; Iyer et al., 2019; Kavusan & Frankort, 2019). Drawing upon insights from research on technological search (Rosenkopf & Nerkar, 2001; Rothaermel & Alexandre, 2009), we offer new empirical evidence in support of a broader range of locus of search choices proposed in the original BTOF (Cyert & March, 1963): Our findings show that organizations respond to performance feedback with changes in the locus of search along not only the technological dimension, which aligns with the concept of familiarity, but also along the organizational dimension, which focuses on the locus relative to firm boundaries.

Our findings further extend research on self-enhancement and learning from performance feedback in two distinct ways. First, the available evidence pointing to self-enhancing responses to performance feedback comes from studies at the organizational level of analysis (Blagoeva et al., 2020; Lucas et al., 2018) and at the individual level (Audia & Brion, 2007; Audia, Brion, & Greve, 2015). Previous work has not directly addressed self-enhancement in the context of subunits subject to accountability pressures arising from hierarchical control that may eliminate the discretion necessary to form self-enhancing assessments of performance. Indeed, Jordan and Audia (2012) identified accountability pressures as a condition that deters a self-enhancement orientation. While more empirical work is needed to tackle this issue (Audia & Greve, 2021), our findings provide rare evidence of self-enhancement in the context of subunit activities.

Second, and more importantly, we show a new modality by which self-enhancement may influence response to performance feedback. While previous work on learning from performance emphasizes reduced responsiveness to low performance as a key manifestation of self-enhancement (e.g., Audia & Brion, 2007; Joseph & Gaba, 2015), we report a different and so far unexplored effect when considering multiple goals whereby self-enhancement directs locus of search choices and related responses to inconsistent performance signals towards less distal (more local) technological and organizational domains. In this way, our theoretical and empirical analyses indicate that a self-enhancing response may not only mean less change, as previous research has suggested, but could also mean guiding decision-makers’ search to (less distal) solutions that reinforce their image of competence. The ambiguity generated by performance inconsistency allows organizations to assess and reprioritize their locus of search choices, instead of only changing emphasis from the poor-performing primary goal to the secondary goal that shows high performance, and thus still continue solution search but towards more familiar and less risky locations. In other words, performance feedback along multiple goals and self-enhancement are not only important in triggering more or less search but also in affecting the direction of search among alternatives, an important aspect of firm responses to performance feedback that has received less attention in the literature but is central to understanding firm responses (Keil, Posen, & Workiewicz, 2022).

Taken together, our arguments and results indicate that self-enhancement may play an even more central role in how organizations and their decision-makers respond to performance feedback, suggesting the need for additional theoretical and empirical research on this construct.

### Limitations and Future Research

Some limitations to our research must be acknowledged. Although the strength of our empirical approach is the ability to track a large number of drug candidates for an important group of incumbent pharmaceutical companies across an entire multiyear clinical trials pipeline, this approach did not allow us to collect detailed information on individual firms, such as the full details of the R&D process (beyond the clinical trials) or the decision-making details for individual projects. Future research should therefore attempt to extend our study through careful, in-depth, qualitative work that could further elucidate how organizations process information from the performance of a portfolio of projects or even individual projects and, in turn, use this information to steer the overall R&D process.

A second limitation of our study is that we began the examination of the R&D process after the patenting of new drug candidates. The performance feedback we investigated in this study may also have implications for the management of activities that precede the selection of patented molecules for preclinical trials. However, systematic information about these basic research and scientific activities is not publicly available and is therefore difficult to collect longitudinally for a large number of firms. Qualitative research may thus be useful to extend the investigation of the effects of performance feedback even earlier in the R&D process and to examine how performance at the late stages affects decision-making in these very early R&D stages.

## Notes

1. While self-enhancement implies that decision-makers prioritize their self-image in decision-making, this does not automatically imply that decisions made with a self-enhancement motive are negative for organizational performance, as both Jordan and Audia (2012) and the recent paper by Levinthal and Rerup (2021) suggest.

2. To minimize potential survivor bias, we sampled pharmaceutical companies at the beginning of the study period (i.e., in 1993).

3. Results were nearly identical if in the calculation of the historical aspirations we weighted recent performance more heavily or if we simply used the previous year's performance as a proxy. Also, our results were robust to any a_2_ greater than 0.35 and lower than 0.96.

4. We also note that the same analysis (i.e., with the count dependent variables) using the OLS panel data provided similar results.

## Appendix

### List of Disease Areas

Alimentary/Metabolic

Anti-infective

Anticancer

Antiparasitic

Blood and Clotting

Cardiovascular

Dermatological

Genitourinary (including sex hormones)

Hormonal (excluding sex hormones)

Immunological

Miscellaneous

Musculoskeletal

Neurological

Respiratory
